# Impact of the downstream myocardial mass on values of coronary microvascular resistance

**DOI:** 10.14814/phy2.15503

**Published:** 2022-11-02

**Authors:** Tadashi Murai, Hiroyuki Hikita, Tim P. van de Hoef, Yoshinori Kanno, Fumiyuki Abe, Keiichi Hishikari, Munehiro Iiya, Naruhiko Ito, Hiroshi Yoshikawa, Hirotaka Yano, Wataru Tsuno, Atsushi Takahashi, Taishi Yonetsu, Tsunekazu Kakuta, Tetsuo Sasano

**Affiliations:** ^1^ Cardiovascular Center Yokosuka Kyosai Hospital Yokosuka Japan; ^2^ Heart Center Amsterdam UMC Amsterdam The Netherlands; ^3^ Department of Cardiovascular Medicine Tokyo Medical Dental University Hospital Tokyo Japan; ^4^ Department of Cardiology Ome Municipal General Hospital Tokyo Japan; ^5^ Department of Cardiovascular Medicine Tsuchiura Kyodo General Hospital Tsuchiura Japan

**Keywords:** coronary blood flow, coronary microvascular resistance, coronary physiology, myocardial mass

## Abstract

The assessment of hyperemic microvascular resistance (HMR) may be dependent on the assessment location in the coronary artery and the amount of partial myocardial mass (PMM) distal to the assessment locations. The aim of this study was to investigate the differences in HMR values between the distal and proximal sites in the same coronary arteries as well as the relationship between HMR and PMM. Twenty‐nine vessels from 26 patients who had undergone intracoronary physiological assessments including Doppler flow velocity at the distal third part and the proximal third part in the same vessels were assessed. The mean values of HMR and PMM at the distal sites were 2.08 ± 0.75 mmHg/cm/sec and 22.2 ± 10.4 g, respectively. At the proximal sites, the values of HMR and PMM were 1.19 ± 0.33 mmHg/cm/sec and 59.9 ± 18.3 g, respectively. All HMR values at the distal sites were significantly higher than those at the proximal sites (*p* < 0.001). Smaller PMM at the distal sites was significantly associated with higher HMR (*r* = −0.544, *p* = 0.002) and was the strongest factor affecting the HMR values (*p* = 0.009), while this relationship was not observed at the proximal sites (*r* = −0.262, *p* = 0.17). The impact of PMM on HMR was diminished at assessment locations where PMM was greater than 35 g. In conclusion, a small amount of downstream myocardial mass could be related to high HMR values. The assessment location around the proximal coronary artery with over 35 g of myocardium would be appropriate to assess HMR because it minimizes the influence of the assessment location.

## Graphical abstract

Hyperemic microvascular resistance (HMR) could theoretically be influenced by downstream myocardial mass, and this study confirmed that a small amount of downstream myocardium could be related to the high values of Doppler flow velocity‐based HMR. The assessments of HMR at the proximal coronary artery, supplying at least 35 g of myocardium, mitigates the impact of myocardial mass on HMR values. This study indicates a potential solution for the issue regarding the assessment location of HMR.

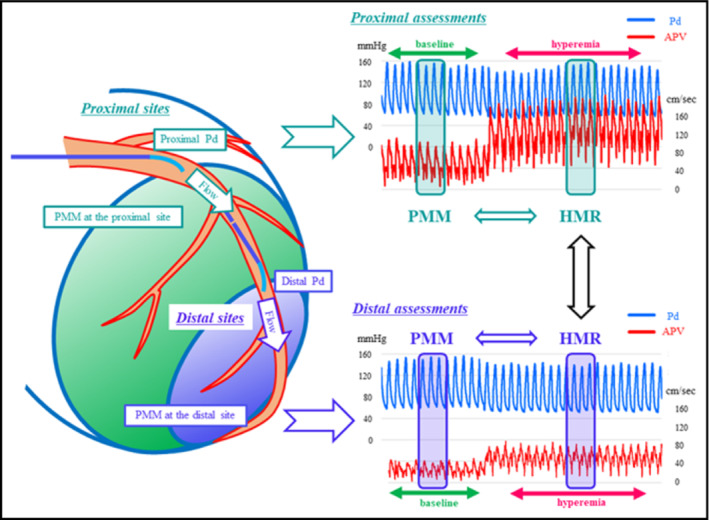

## 1 INTRODUCTION

Indices of coronary microvascular resistance at hyperemia have been introduced as specific markers of microvascular function, and are calculated from invasive measurements of distal coronary pressure (Pd) and coronary blood flow (Chamuleau et al., 2003; Fearon et al., 2003; Siebes et al., 2004). Echavarría‐Pinto et al. reported an important feature of thermodilution‐derived hyperemic microvascular resistance (index of microcirculatory resistance: IMR): that the values of IMR are influenced by the amount of downstream myocardial mass (Echavarría‐Pinto et al., 2017). This caveat of IMR can be explained by the fact that coronary flow decreases after branching of the coronary artery, while the driving pressure remains virtually unchanged (Di Mario & Serruys, 1995; Seiler, 1997). This unbalanced change between coronary blood flow and pressure leads to an increase in the calculated amount of “microvascular resistance” value in the smaller areas of downstream myocardial mass.

**FIGURE 1:**
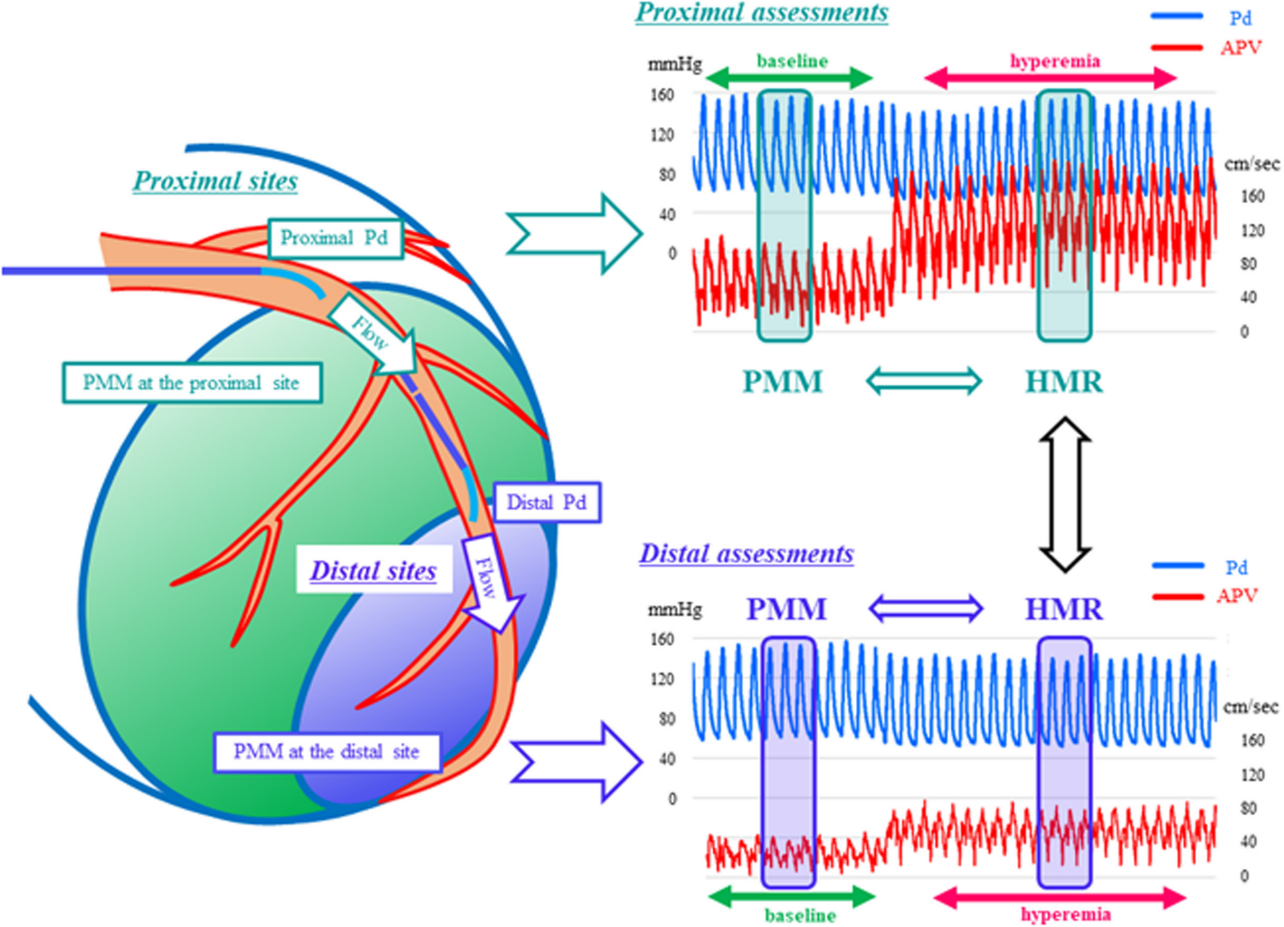
The schema of this study. Distal coronary pressure and Doppler flow velocity were measured at the distal third and proximal third parts of the same coronary artery. The values of HMR at the distal and proximal sites were compared, and the relationship between HMR and PMM at each site was investigated. APV, mean average peak flow velocity; HMR, hyperemic microvascular resistance; Pd, mean proximal coronary distal pressure; PMM, partial myocardial mass distal to the assessment location

Compared to coronary flow volume‐based microvascular resistance like IMR, Doppler flow velocity‐based hyperemic microvascular resistance (HMR) is theoretically less influenced by downstream myocardial mass, because flow velocity is less changed after blanching compared with flow volume (Di Mario & Serruys, 1995; Seiler, 1997). However, the impact of downstream myocardial mass on HMR has not been specifically investigated, and the spatial changes in HMR in the same coronary arteries have not been assessed. We aimed to investigate the difference in HMR between the proximal and distal sites in the same coronary arteries and the relationship between HMR and partial myocardial mass distal to the assessment location (PMM) estimated by intra‐coronary physiological assessment (Murai et al., 2019; Figure 1).

## 2 METHODS

### 2.1 Study design and patient population

This prospective study was conducted at Yokosuka Kyosai Hospital. Among patients who were scheduled for coronary angiography and intra‐coronary physiological assessment as a part of clinical practice between April 2020 and July 2020 because of suspected myocardial ischemia, those patients who had a feasible coronary artery for physiological assessments in both distal and proximal sites of the same coronary artery criteria were enrolled in this study. The exclusion criteria were prior coronary artery bypass grafting, left main coronary artery disease, prior myocardial infarction in the perfusion territory of the investigated coronary artery, unstable heart failure, significant valvular disease, extremely tortuous vessels, and significantly reduced systolic left ventricular function (ejection fraction <50%). Vessels of patients with prior myocardial infarction in a non‐investigated coronary artery could be included. For the purpose of this study, lesions with angiographically severe stenosis (% diameter stenosis >75%) were also excluded because of the possible existence of significant collateral coronary flow, which is less suitable for selective assessment of microvascular resistance and downstream myocardial mass. The study was approved by the Human Research Ethics Committee of Yokosuka Kyosai Hospital, and all patients provided written informed consent.

### 2.2 Sample size estimates

The number of patients included in this study is based on the estimation of sample size needed to identify a statistically significant difference in HMR values between the distal and proximal assessments. On the basis of the previous study (Murai et al., 2019), the primary endpoint chosen to evaluate the hypothesis is the individual difference in HMR between the distal and proximal sites, that is 20% decrease in HMR from the distal sites to the proximal sites. Using an estimate of an expected difference in HMR from an assumed HMR at the distal sites of 2.0 to HMR at the proximal sites of 1.6 with an alpha error of 0.05, a power 80%, and a standard deviation of 0.6, we estimated that the required sample size is at least 20 paired samples to detect the difference of HMR. To take the expected exclusion rate of the paired assessments on the basis of the previous study (36%) (Johnson et al., 2021), we expected a total of at least 32 paired samples would be required in the present study.

### 2.3 Coronary angiographic and physiological assessments

Before angiography, nitroglycerin (200–300 μg) was administered to the coronary artery. Coronary angiography was performed using standard techniques. After the operator confirmed the absence of angiographic exclusion criteria, each eligible vessel underwent physiological assessment using a 0.014‐inch dual sensor‐equipped guidewire (Combowire; Philips‐Volcano) to obtain simultaneous coronary pressure and flow velocity data. Intra‐coronary physiological assessments were performed at the distal third part and the proximal third part of the same vessel. The corresponding Doppler flow sensing positions were recorded on cine angiograms. Offline quantitative coronary angiography analyses were performed to measure the vessel diameter at the Doppler flow sensing position (5 mm distal to the sensor position) using validated software (QAngio® XA, Medis). The vessel diameter was assessed by two different directional angiograms at the end diastolic phase; their mean value was calculated and used for further calculations. Intra‐coronary physiological assessment was performed under stable conditions at rest and during hyperemia. Hyperemia was induced by intracoronary administration of adenosine (100 μg). Physiological data were extracted from the digital archive (ComboMap, Philips‐Volcano), and analyzed offline for the measurement of distal coronary pressure (Pd) and the average peak flow velocity (APV) at both resting and hyperemic status. For the additional analysis of resting physiological data, the corresponding systolic blood pressure (sBP) and mean heart rate (HR) were also obtained from raw digitized data.

### 2.4 Calculation of the physiological indices

PMM was assessed according to the method described previously (Murai et al., 2019). In brief, based on the flow continuity principle between coronary flow supply and myocardial demand in the resting condition, PMM can be assessed using measurable resting physiological parameters (baseline APV [bAPV], sBP, HR), and the vessel diameter (D) at the Doppler sensing position. HMR was calculated as Pd at hyperemia divided by hyperemic APV (hAPV). Hyperemic stenosis resistance of epicardial coronary artery (HSR) and total coronary vascular resistance (TVR) were also assessed. Each formula of the indices is as follows.

$${\mathrm{PMM}}_{\left(g\right)}=\text{bAPV}\times D^{2}\times \pi /\left(1.24\times 10^{-3}\times {\mathrm{HR}}_{\text{rest}}\times {\mathrm{sBP}}_{\text{rest}}+1.6\right)$$

$$\mathrm{HMR}\left(\text{mmHg}/\mathrm{cm}/\sec\right)={\mathrm{Pd}}_{\text{hyperaemia}}/\text{hAPV}$$

$$\mathrm{HSR}\left(\text{mmHg}/\mathrm{cm}/\sec\right)=\left({\mathrm{Pa}}_{\text{hyperaemia}}-{\mathrm{Pd}}_{\text{hyperaemia}}\right)/\text{hAPV}$$

$$\mathrm{TVR}\left(\text{mmHg}/\mathrm{cm}/\sec\right)={\mathrm{Pa}}_{\text{hyperaemia}}/\text{hAPV}$$

### 2.5 Statistical analysis

Data were analyzed on a per‐patient basis for clinical characteristics and on a per‐region basis for the other calculations. Normality of the distribution of the values was assessed using Shapiro–Wilk statistics; and the homogeneity of variances was assessed using Levene's test. Continuous variables are expressed as the mean ± standard deviation for the normally distributed variables and as the median values with the first and third quartiles (Q1, Q3) for the non‐normally distributed variables. Categorical variables are presented as counts and percentages. The physiological parameters and indices at the distal and proximal sites were compared using paired t‐tests for normally distributed variables and Wilcoxon tests for non‐normally distributed variables. The relationships between HMR and PMM were assessed using Pearson's correlation for normally distributed variables and Spearman's rank correlation for non‐normally distributed variables. The determinant factors of HMR at the distal and proximal sites were evaluated using univariate and multivariate linear regression analyses. Among all the variables, including patient characteristics and angiographic findings, the variables associated with HMR values showing *p* value ≤0.10 by univariate analysis in any one of the assessment sites were entered into the final multivariate model. Statistical analysis was performed using SPSS (version 22.0; SPSS, Inc.) and R version 4.1.2. Statistical significance was set at *p* < 0.05.

## 3 RESULTS

### 3.1 Patient characteristics

The initial study population was comprised of 36 vessels from 33 patients who underwent intra‐coronary physiological assessments. After careful assessment of each myocardial region, 7 vessels from 7 patients were excluded from the analysis because of inadequate data quality. The remaining 29 vessels from 26 patients were included in the analysis. The mean diameter stenosis and fractional flow reserve (FFR) values were 37.4 ± 13.3%, and 0.84 ± 0.09, respectively. Complete baseline characteristics are presented in Table 1.

**TABLE 1:** Baseline characteristics

Clinical characteristics	*n* = 26 patients
Patient characteristics	
Age, years	73.3 ± 8.1
Male, n (%)	22 (84.6)
Height, cm	161.9 ± 8.0
Weight, kg	64.4 ± 13.6
BMI, kg/m^2^	24.4 ± 4.1
EF, %	66 (62–71)
Coronary risk factors	
Hypertension, n (%)	16 (61.5)
Hyperlipidemia, n (%)	19 (73.1)
Diabetes Mellitus, n (%)	7 (26.9)
Smoking history, n (%)	15 (57.7)
Medication, n (%)	
Aspirin	15 (57.7)
ACE‐I or ARB	15 (57.7)
β‐blocker	15 (57.7)
CCB	13 (50.0)
Statin	19 (73.1)
Laboratory data	
WBC, /μl	5800 (5300–7300)
Hemoglobin, g/dl	13.6 ± 1.3
Albumin, g/dl	4.3 ± 0.3
Creatinine, mg/dl	0.93 (0.81–1.04)
eGFR, ml/min/1.73m^2^	58.2 (53.5–65.9)
LDL‐C, mg/dl	79 (70–102)
HDL‐C, mg/dl	52 ± 14
TG, mg/dl	120 (90–178)
HbA1c, %	6.2 (5.7–7.1)
BNP, pg/ml	42 (20–119)
Angiographic characteristics	*n* = 29 vessels
Target regions	
LAD, n (%)	23 (79.3)
LCX, n (%)	3 (10.3)
RCA, n (%)	3 (10.3)
QCA lesion assessments	
MLD, mm	1.66 ± 0.51
RD, mm	2.64 ± 0.58
% DS, %	37.4 ± 13.3
Lesion length, mm	7.4 (5.8–9.8)

Abbreviations: ACE‐I indicates angiotensin‐converting enzyme inhibitor; ARB, angiotensin receptor blocker; BMI, body mass index; BNP, B‐type natriuretic peptide; CCB, calcium channel blocker; DS, diameter stenosis; EF, ejection fraction; eGFR, estimated glomerular filtration rate; HbA1c, glycated hemoglobin; HDL‐C, High‐density lipoprotein cholesterol; LAD, left anterior descending coronary artery; LCX, left circumflex artery; LDL‐C, Low‐density lipoprotein cholesterol; MLD, minimal lumen diameter; QCA, quantitative coronary angiography; RCA, right coronary artery; RD, reference diameter; TG, Triglyceride; WBC, white blood cell.

### 3.2 The differences of HMR and PMM between the distal and proximal sites

**FIGURE 2:**
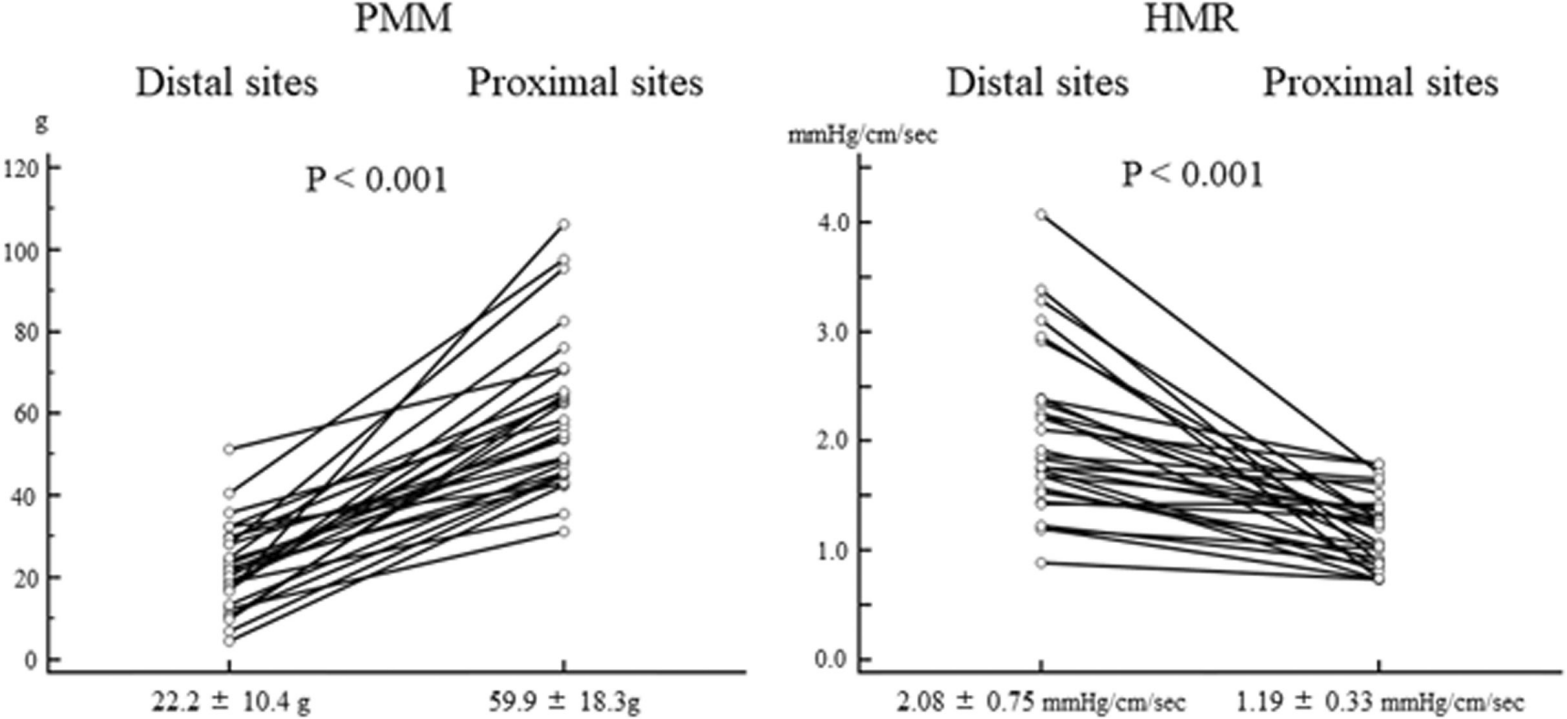
The comparison of PMM and HMR between the distal and proximal sites. PMM and HMR values at the distal and proximal sites were compared using a paired t‐test. All the HMR values at distal sites were greater than those at the proximal sites. HMR, hyperemic microvascular resistance; PMM, partial myocardial mass distal to the assessment location

Table 2 shows the physiological parameters at the distal and proximal sites. The mean values of PMM were 22.1 ± 10.4 g at the distal sites and 59.9 ± 18.3 g at the proximal sites. Aortic pressure and HR were not significantly different between distal and proximal assessments. All of the values of HMR at the distal sites were higher than those at the proximal sites [mean value of HMR: 2.08 ± 0.75 mmHg/cm/sec at the distal sites versus 1.19 ± 0.33 mmHg/cm/sec at the proximal sites (*p* < 0.001)] and the mean difference in HMR between the distal and proximal sites was 0.89 ± 0.74 mmHg/cm/sec (37.5 ± 21.2% decrease in HMR from the distal sites to the proximal sites). (Figure 2).

**TABLE 2:** Physiological parameters at distal and proximal sites

Parameters and indices	Distal sites	Proximal sites	*p*
Pa at rest, mmHg	86.5 ± 15.1	85.2 ± 13.9	0.29
Pd at rest, mmHg	80.3 ± 15.0	83.3 ± 13.8	0.053
APV at rest, cm/sec	18.7 ± 5.5	31.1 ± 10.3	<0.001
HR at rest, bpm	66.3 ± 9.4	67.8 ± 10.7	0.14
Pa at hyperemia, mmHg	79.6 ± 14.2	77.3 ± 14.1	0.13
Pd at hyperemia, mmHg	66.6 ± 13.3	73.2 ± 14.2	0.001
APV at hyperemia, cm/sec	36.0 ± 13.9	66.6 ± 24.8	<0.001
HR at hyperemia, bpm	66.3 ± 9.5	65.9 ± 10.0	0.66
FFR	0.86 (0.78–0.90)	0.97 (0.91–0.99)	<0.001
CFR	1.92 ± 0.44	2.23 ± 0.71	0.002
HMR, mmHg/cm/sec	2.08 ± 0.75	1.19 ± 0.33	<0.001
HSR, mmHg/cm/sec	0.31 (0.16–0.62)	0.03 (0.01–0.09)	<0.001
TVR, mmHg/cm/sec	2.15 (1.83–2.91)	1.28 (0.96–1.46)	<0.001
PMM, g	22.1 ± 10.4	59.9 ± 18.3	<0.001

Abbreviations: APV indicates average peak coronary flow velocity; angiography; CFR, coronary flow reserve; FFR, Fractional flow reserve; HMR, hyperemic microvascular resistance; HR, heart rate; HSR, Hyperemic stenosis resistance of epicardial coronary artery; Pa, mean aortic pressure; Pd, mean distal coronary pressure; PMM, partial myocardial mass distal to the assessment locations; TVR, total coronary vascular resistance.

### 3.3 The relationships between HMR and PMM

**FIGURE 3:**
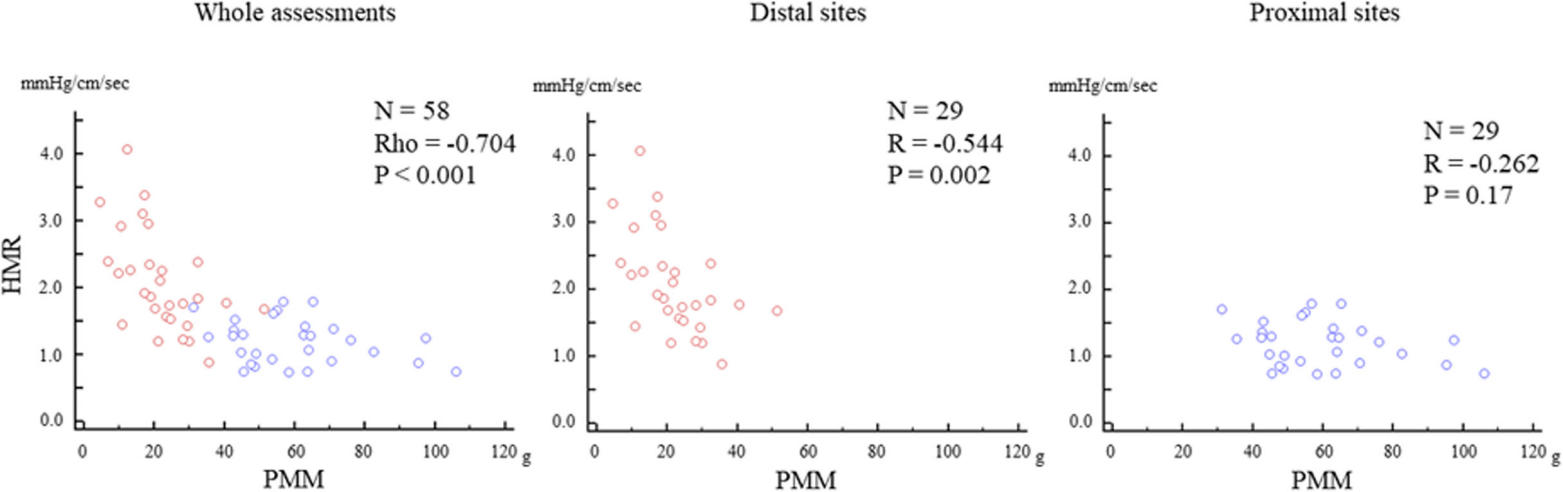
The relationships of HMR with PMM between the distal sites and proximal sites. The relationships between HMR and PMM values were investigated using Spearman's rank correlation for whole assessments and Pearson's correlation for the distal and proximal sites. Red circles represent the distal assessments and blue circles represent those at the proximal sites. HMR, hyperemic microvascular resistance; PMM, partial myocardial mass distal to the assessment location

Figure 3 shows the overall relationships between HMR and PMM, as well as for proximal and distal assessment locations separately. Across the whole range of measurements, a smaller PMM was significantly associated with higher HMR (Spearman's rho = −0.704, *p* < 0.001). This relationship was also observed at the distal sites (*r* = −0.544, *p* = 0.002). In contrast, there was no significant relationship between HMR and PMM at the proximal sites (*r* = −0.262, *p* = 0.17). A PMM threshold of 35 g best delineated assessment locations where PMM significantly affected HMR values (PMM <35 g: *r* = −0.563, *p* = 0.002) versus locations where PMM did not significantly affect HMR values (PMM ≥35 g: *r* = −0.204, *p* = 0.27). The variables associated with HMR which showed *p* value ≤0.10 by univariate linear regression analysis in one of any assessment sites were listed in Table 3. Multivariate linear regression analysis showed that PMM was the strongest factor determining HMR values at the distal sites (*p* = 0.009), while PMM was not a significant factor at the proximal sites.

**TABLE 3:** Univariate and multivariate linear regression analyses to predict HMR values at distal and proximal sites

	Distal sites						Proximal sites					
	Univariate linear regression			Multivariate linear regression			Univariate linear regression			Multivariate linear regression		
	β	95% CI	*p*	β	95% CI	*p*	β	95% CI	*p*	β	95% CI	*p*
HT	0.179	−0.404–0.761	0.53				−0.246	−0.486–−0.006	0.045			
ACEI/ARB	−0.033	−0.625–0.560	0.91				−0.303	−0.536–−0.070	0.013	−0.303	−0.536–−0.070	0.013
CCB	0.516	−0.031–1.063	0.063				0.066	−0.190–0.323	0.60			
HDL‐C	0.025	0.006–0.044	0.014				0.005	−0.004–0.014	0.29			
TG	0.002	−0.003–0.007	0.51				0.002	0.000–0.004	0.077			
Albumin	−0.600	−1.610–0.410	0.23				0.389	−0.043–0.820	0.076			
MLD	−0.649	−1.169–−0.129	0.016				0.103	−0.150–0.355	0.41			
% DS	0.027	0.008–0.047	0.007	0.020	0.002–0.039	0.028	−0.001	−0.011–0.009	0.79			
PMM	−0.039	−0.063–−0.015	0.002	−0.032	−0.055–−0.009	0.009	−0.005	−0.012–0.002	0.17			

Abbreviations: ACE‐I indicates angiotensin‐converting enzyme inhibitor; ARB, angiotensin receptor blocker; CCB, calcium channel blocker; DS, diameter stenosis; HDL‐C, High‐density lipoprotein cholesterol; HT, hypertension; MLD, minimal lumen diameter; PMM, partial myocardial mass distal to the assessment locations; TG, Triglyceride.

## 4 DISCUSSION

Our major findings were that the HMR values can be influenced by the assessment location in the coronary arteries. A smaller amount of downstream myocardial mass significantly contributed to higher values of HMR at the distal site, while the relationship was diminished at the proximal site where the value of downstream myocardial mass was over 30 g. A PMM threshold of 35 g best delineated assessment locations where PMM significantly affected HMR values versus locations where PMM did not significantly affect HMR values.

### 4.1 The issue of “coronary microvascular resistance” as an assessment of microvascular function

Microvascular dysfunction has been recognized as one of the determinant factors of clinical outcomes in patients both with or without coronary artery disease (Crea et al., 2014; Crea et al., 2017; Lanza et al., 2013; Marzilli et al., 2012; Pepine et al., 2010). Indices of microvascular resistance were introduced to selectively assess microvascular function, including in patients with coronary artery disease, and play an increasingly important role in the diagnosis of chest pain syndromes (Ford et al., 2020; Mejía‐Rentería et al., 2017). Because some diagnostic algorithms solely apply microvascular resistance measurements in the diagnosis of microvascular dysfunction, the methodological concerns for such resistance calculations deserve particular attention.

In line with previous findings in coronary thermodilution‐derived microvascular resistance measurements (Echavarría‐Pinto et al., 2017), our data support the dependence of microvascular resistance calculated from Pd and any type of coronary flow assessment on the amount of subtended myocardial mass, especially in small myocardial territories. This fundamental issue of HMR would indicate that microvascular resistance values cannot be compared between different assessment locations or different patients. This issue might explain the lack of long‐term clinical evidence of microvascular resistance, regardless of the measurement technique, in patients with chronic coronary syndromes. Lesions at the distal sites of coronary arteries are unlikely to be associated with the occurrence of adverse clinical events, though such lesions are more likely to show high microvascular resistance values simply due to the small subtended myocardial mass (Adjedj et al., 2016; Hachamovitch, 2015). In other words, the vessels diagnosed with microvascular dysfunction by contemporary microvascular resistance thresholds are those with high HMR. However, these high values can be due not only to true impairment of microvascular conductance, but also simply to the distal assessment location with small subtended myocardial mass, in which adverse events are less likely to occur.

### 4.2 The appropriate assessment location for HMR to minimize the impact of downstream myocardial mass

In current clinical practice, sensor locations for physiological assessment using coronary pressure measurements, like FFR, are generally decided according to the location of epicardial coronary stenoses, ensuring assessment at least 3 vessel diameters distal to the lesion but frequently sufficiently distal in the coronary architecture to appreciate pressure loss along the length of the coronary artery. Therefore, the sensor positions are frequently located at very distal sites. However, the appropriate sensor location for the assessment of microvascular resistance has not been defined. According to our results and supported by data on coronary thermodilution (Echavarría‐Pinto et al., 2017), the downstream myocardium should be taken into consideration for the interpretation of microvascular resistance values, where the diagnostic relevance of microvascular resistance measurements in small myocardial territories is questionable.

**FIGURE 4:**
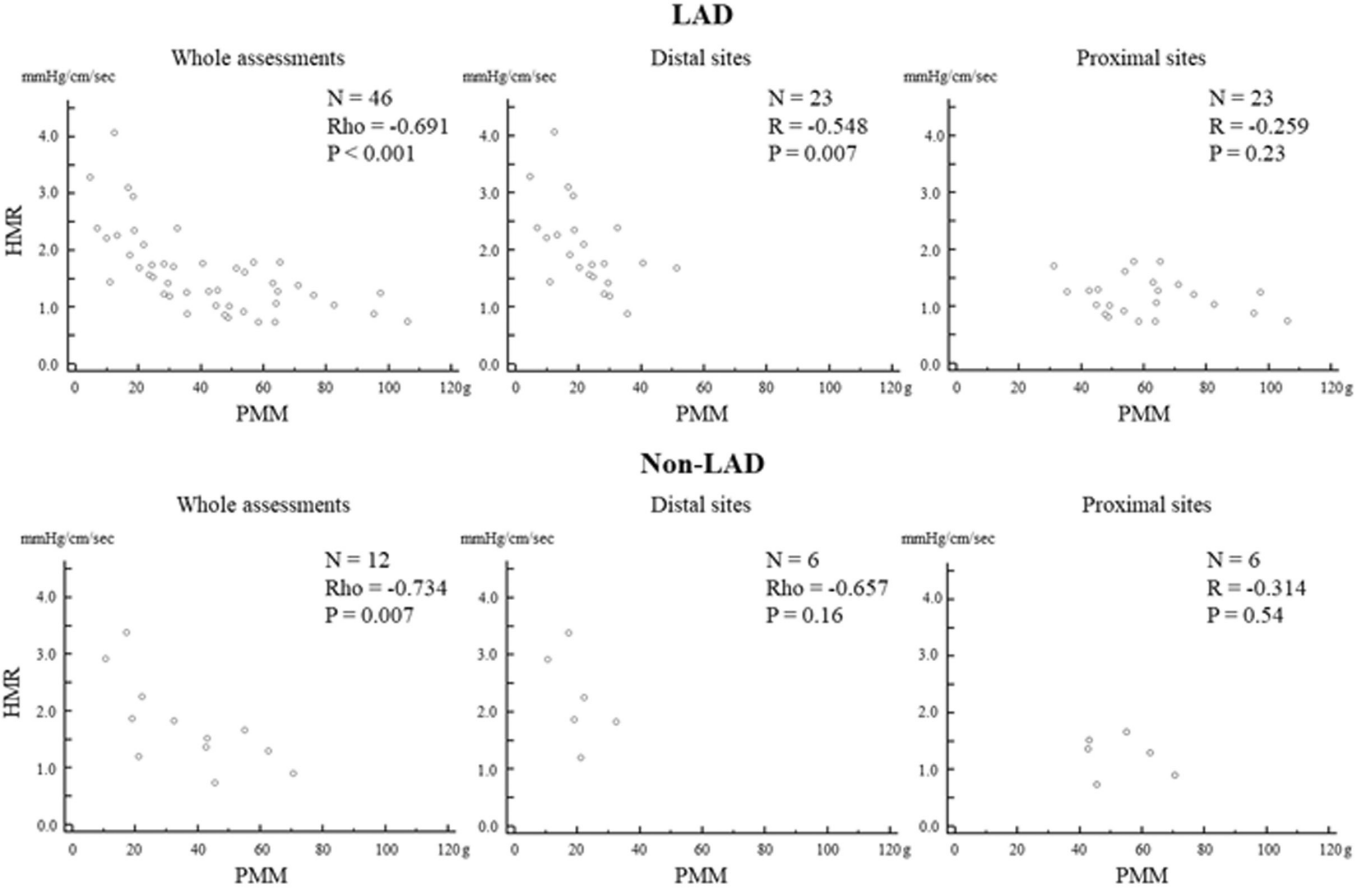
The differences of the relationships between HMR and PMM in LAD and non‐LAD assessments. HMR, hyperemic microvascular resistance; LAD, left anterior descending artery; PMM, partial myocardial mass distal to the assessment location

This study showed that PMM had a strong impact on HMR in the distal sites, although the relationship became insignificant in the proximal sites. This suggests that the influence of PMM on HMR values could be ignored in locations with larger amounts of downstream myocardium. In this study, the thresholds of PMM less than 35 g showed the group with PMM over the threshold had the statistically significant correlation between HMR and PMM, while the impact of PMM on HMR was diminished at assessment locations where PMM was greater than 35 g (PMM ≥35 g: *N* = 31, *R* ^2^ = 0.042, *p* = 0.27; PMM ≥32 g: *N* = 33, *R* ^2^ = 0.12, *p* = 0.047; PMM ≥30 g, *N* = 35, *R* ^2^ = 0.13, *p* = 0.034). Namely, an assessment location around the proximal part of the coronary artery with over 35 g of myocardium would be better for assessing HMR due to minimizing the influence of subtended myocardium on the assessment of HMR. In left anterior descending artery (LAD) assessments, only one assessment of the proximal third part of the LAD measured 31 g, and the other 22 assessments (95.6%) had over 35 g of PMM. Therefore, proximal LAD assessments would be appropriate for assessing HMR. This finding is quite useful because PMM is not generally assessed in current clinical practice. On the other hand, all PMM values in non‐LAD proximal assessments of this study were over 35 g (the median value 50.2 g [Q1‐Q3: 43.1–62.6 g]). This large amount of PMM in these areas might be due to a selection bias of the present study, because only the accessible vessels which were large enough to assess Doppler flow in the distal sites were included in this study. Although a similar relationship between HMR and PMM was observed in non‐LAD assessments (Figure 4), the appropriate assessment location of HMR in non‐LAD assessments remains inconclusive, especially in the small vessels. In addition, it is still unclear to what extent these results apply to other indices of microvascular resistance like IMR. To optimize the assessment of “microvascular resistance” for coronary microvascular function, however, further investigations are required to document optimal sensor position for microvascular resistance measurements.

## 5 STUDY LIMITATIONS

This study included a small number of patients from a single center. Hence, it was not possible to perform sub‐analyses such as vessel differences and other determinant factors of HMR, especially in the proximal sites. The assessed vessels had small or intermediate stenotic lesions between the distal and proximal sites (median values of change in FFR from distal to proximal sites: 0.08 [IQR: 0.05–0.14]). The difference in HMR values between the distal and proximal sites might be affected by the difference of epicardial stenotic resistance and not partial myocardial mass distal to the assessment location. In this study, HSR, which is a representative index of epicardial stenotic resistance, was also higher in the distal sites than that in the proximal sites (median HSR: 0.31 mmHg/cm/sec for the distal sites vs. 0.03 mmHg/cm/sec for the proximal sites, *p* < 0.001) (Table 2). Hence, TVR, which is the total amount of HSR and HMR, was significantly higher in the distal sites than in the proximal sites, although TVR must be consistent irrespective of the proximal or distal sensor position if there is no influence of assessment location on these indices (median TVR: 2.15 mmHg/cm/sec for the distal sites vs. 1.28 mmHg/cm/sec for the proximal sites, *p* < 0.001). These results suggest that not only HMR but also HSR and even TVR could be influenced by the assessment locations because of the unbalanced change between coronary blood flow and pressure. This study was conducted in a single center and all participants were Japanese. Different populations with different body sizes and myocardial volume might affect the results. However, our findings based on the serial changes in HMR in the same vessels would indicate the common principle of HMR. The assessment of PMM is based on the assumption of laminar flow (Ferrari et al., 2006). This assumption is warranted under a critical Reynolds number of 500. The mean Reynolds numbers at hyperemia in the proximal sites were 240 ± 81, and the range was 115–443. Therefore, there was no vessel over the critical Reynolds number, and this assumption is warranted in this study. When further investigations in larger vessels are conducted, this limitation should be taken into account when interpreting the results. Finally, this study does not provide prognostic efficacy of HMR at either proximal and distal assessments. Therefore, this study does not clarify which assessment location of HMR has prognostic implications. Furthermore, lack of the comparison with other independent reference tests for microcirculation is another major limitation of the present study. Further investigations are needed to clarify the appropriate location for HMR assessment.

## 6 CONCLUSION

A small amount of downstream myocardial mass could be related to high HMR values, while the relationship was diminished at the proximal site of the coronary artery with a large amount of myocardium. An assessment location with over 35 g of myocardium would be appropriate to assess HMR because it minimizes the influence of the assessment location. This study indicates a solution to the issue regarding the ideal assessment location to calculate HMR.

## FUNDING INFORMATION

None.

## CONFLICT OF INTEREST

T.M. has received fees from Abbott Vascular Japan and Phillips‐Volcano Japan for educational events and T.P.H. have served as a speaker at educational events organized by Philips‐Volcano, St. Jude Medical, and Boston Scientific, and received institutional research grants from Philips‐Volcano.
